# Prospective randomized controlled trial on comparison of standard CO_2_ pressure pneumoperitoneum insufflator versus AirSeal®

**DOI:** 10.1007/s00464-020-07846-4

**Published:** 2020-08-07

**Authors:** Rosalia Luketina, Theodore L. H. Luketina, Stavros A. Antoniou, Gernot Köhler, Sören Könneker, Lisa Manzenreiter, Helwig Wundsam, Oliver Owen Koch, Michael Knauer, Klaus Emmanuel

**Affiliations:** 1 Department Hand, Plastic, Reconstructive and Burn Surgery, BG Trauma Center Tuebingen, Eberhard Karls University Tuebingen, Schnarrenbergstr. 95, 72076 Tuebingen, Germany; 2 Department of Anaesthesia & Intensive Care Medicine, Cantonal Hospital of Muensterlingen, Muensterlingen, Switzerland; 3 Department of Surgery, School of Medicine, European University Cyprus, Nicosia, Cyprus; 4 Department of General and Visceral Surgery, Congregation Hospital (Sisters of Charity), Ordensklinikum Linz, Austria; 5 Department of Plastic, Aesthetic, Hand and Reconstructive Surgery, Hannover Medical School, Hanover, Germany; 6 Department of Surgery, Paracelsus Medical University, Salzburg, Austria; 7 Breast Center Ostschweiz, Schuppistrasse 10, 9016 St. Gallen, Switzerland

**Keywords:** Laparoscopic surgery, AirSeal®, Pneumoperitoneum, Randomized, Cholecystectomy, Hernia

## Abstract

**Background:**

AirSeal® is a valve-free insufflation system that enables a stable pneumoperitoneum with continuous smoke evacuation and CO_2_ recirculation during laparoscopic surgery. Comparative evidence on the use of AirSeal® and standard CO_2_ insufflator in laparoscopic general surgery procedures is scarce. The aim of this study was to compare surgical outcomes between AirSeal® and standard CO_2_ insufflators in patients undergoing the most frequently performed laparoscopic procedures.

**Methods:**

One hundred and ninety-eight patients undergoing elective laparoscopic cholecystectomy, colorectal surgery and hernia repair were randomized to either AirSeal® (group A) or standard pressure CO_2_ insufflator (group S). The primary endpoints were operative time and level of postoperative shoulder tip pain (Visual Analog Scale). Secondary outcomes included Clavien–Dindo grade complications, surgical side effect and length of hospital stay.

**Results:**

Patients were randomized to either group A (*n* = 101) or group S (*n* = 97) and were analyzed by intention-to-treat. There was no significant difference in mean operative time between the groups (median [IQR]; 71 min [56–94] in group A vs. 69 min [52–93] in group S; *p* = 0.434). Shoulder tip pain levels were significantly lower in group S (VAS 0 [0–3] in group S vs. 2 [0–4] in group A; *p* = 0.001). There was no significant difference in complications, surgical side effects (subcutaneous emphysema was not observed in any group) and length of hospital stay.

**Conclusion:**

This randomized controlled trial showed that using the AirSeal® system did not reduce operative time and was associated with a higher postoperative shoulder tip pain compared to standard CO_2_ insufflator for short elective surgeries.

ClinicalTrials.gov (NCT01740011).

Laparoscopic surgery has become widely adopted in managing gastrointestinal, gynecologic, and urologic diseases [1]. Adequate exposure of the operative field facilitates technical performance and is a factor that affects duration of surgery and patient safety [2]. Conventional CO_2_ insufflation systems often respond with delay to intraoperative loss of intra-abdominal pressure. The collapse of the abdominal cavity during increased systemic absorption of CO_2_ gas, for example as a result of suction or smoke evacuation, may prolong operation time and can be prevented only with an increase in CO_2_ insufflation pressure. The CO_2_ insufflation and higher abdominal pressure adversely affect patient homeostasis, causing significant changes in cardiovascular and respiratory systems, decreasing perfusion in abdominal organs and blood flow in the inferior vena cava, and posing increased risk of thrombotic disease [3–5].

In addition, shoulder pain is a common complaint following laparoscopic surgery [6, 7]. The origin of shoulder pain is multifactorial and still poorly understood. Possible causes are the irritative effect of CO_2_ [8, 9], peritoneal and diaphragmatic stretching and injury [9] and residual pockets of gas in the abdominal cavity after surgery [10]. AirSeal® (SurgiQuest, Inc., Milford, CT), a novel class of valve and membrane-free insufflation/trocar system, has become available. This system responds immediately to slightest changes in intra-abdominal pressure maintaining a stable-pressure pneumoperitoneum and continuous smoke evacuation even under difficult surgical conditions and constant suction, ensuring adequate visibility. It has been associated with reduced need of CO_2_ insufflation, absorption and elimination [11]. Although the reduction of CO_2_ absorption makes this new insufflation system an attractive alternative to standard insufflation systems, no randomized clinical studies in humans have been performed to demonstrate significant benefits and advantages in laparoscopic general surgery procedures up to now.

We hypothesized that patients operated with AirSeal® have a shorter mean operative time and decreased frequency and intensity of postoperative shoulder tip pain compared with patients undergoing surgery with standard pressure CO_2_ insufflation systems. Therefore, the primary objective was to compare the mean operative time and to study the frequency and intensity of postoperative shoulder tip pain in patients undergoing laparoscopic surgery, i.e., cholecystectomy, colorectal surgery, hernia repair with AirSeal® compared with standard pressure CO_2_ insufflation systems.

## Materials and methods

### Study design

This prospective, randomized clinical trial (RCT) was conducted at the Department of General and Visceral Surgery of Sisters of Charity Hospital Linz (Linz, Austria), a high-volume tertiary care center with experience in advanced laparoscopic surgery. The aim of this study was to compare AirSeal® to a standard CO_2_ insufflator for surgical outcomes in patients who underwent laparoscopic procedures in visceral surgery. The study protocol was approved by the ethical committee of the Hospital of the Sisters of Charity in Linz, a member of the independent Ethics Committee (IEC) of Austria (Study number: 28/12 AirSeal® Trial) and all patients gave written informed consent for participation. The study was conducted in accordance with principles of the Declaration of Helsinki and guidelines for Good Clinical Practice [12]. The study was registered on ClinicalTrials.gov with inscription number NCT01740011. Consecutive patients scheduled to undergo laparoscopic surgery at our department were recruited between January 2013 and January 2014. Results are presented according to the CONSORT statement (Supplemental Digital Content 1, https://links.lww.com/SLA/B352) [13]. The study protocol has been published previously [14].

### Subjects

Consecutive patients scheduled for elective laparoscopic cholecystectomy, colorectal surgery (sigmoid resection) and hernia repair (unilateral/bilateral inguinal hernias or ventral abdominal wall hernias), aged over 18 years, were included in the study. Clinical evaluation and pre-randomization assessment were completed for every patient including the review of eligibility criteria, a signed and dated informed consent, inquiry of relevant past medical history and anesthesiologic preoperative assessment, including American Society of Anesthesiologists (ASA) class. In case of conversion to open surgery, participants were excluded from per-protocol analysis. Exclusion criteria were as follows: previous extensive abdominal surgery, e.g., previous laparotomy with major organ resection; previous urgent/emergency abdominal surgical intervention, immunological disfunction, severe chronic hepatic, renal, pulmonary, or cardiac disease; pregnancy and lactation; and patient’s refusal to participate.

### Randomization

Patients undergoing elective laparoscopic cholecystectomy, colorectal surgery or hernia repair were randomized 1:1 to either laparoscopic surgery with an AirSeal® CO_2_ pressure insufflator (group A) or with a standard CO_2_ pressure insufflator (group S). Randomization was stratified according to type of operation (cholecystectomy, sigmoid resection, hernia-inguinal unilateral/bilateral or ventral hernia). Patients were randomized using a web-based, central randomization and registration system (www.randomizer.at) upon induction of anesthesia in the preoperative area.

### Interventions

All patients were operated in one center by three surgeons, with experience in advanced laparoscopic surgery. A pilot study had previously been carried out with 86 patients in each group for the prior ranking primary endpoint (time of surgery). In this retrospective evaluation, operative time was significantly longer in standard CO_2_ pressure insufflator group compared to AirSeal® CO_2_ pressure insufflator group (68 ± 15 vs. 58 ± 15 min (mean ± SD), *p* = 0.026) in patients undergoing laparoscopic cholecystectomy. All procedures were performed under general anesthesia, and the surgical technique and perioperative care were performed in the following manner. In all patients, access was achieved using four working ports (trocars) for cholecystectomy, as well as sigmoid resection and three working ports for the herniotomy procedure. The use of surgical instruments was standardized and did not differ within the operation groups. Pneumoperitoneum was created using a Veress needle inserted through a small skin incision in the umbilical region. Reverse Trendelenburg position with both arms secured to the sides was used after the induction of pneumoperitoneum in cholecystectomy and Trendelenburg position in inguinal hernia and sigmoid resection procedures. Operative details for laparoscopic ventral hernia repair and transabdominal pre-peritoneal (TAPP) repair have been described previously [15, 16]. Pneumoperitoneum was created using the AirSeal® system CO_2_ pressure insufflator (Surgiquest Inc., Milford, USA insufflator) (group A) or with a standard CO_2_ pressure insufflator (Olympus America Inc. UHI-3, Center Valley, PA, US) (group S) with a CO_2_ flow rate of 2 L/min. Having created a 12 mmHg pneumoperitoneum, the surgeons proceeded to insert trocars. The AirSeal® consists of three devices: Intelligent Flow System (IFS), the AirSeal® trocar, and the AirSeal® Mode Evacuation (ASM-Evac) Tri-lumen Filter Tube Set. The AirSeal® valve-free trocar includes CO_2_ nozzles that act as pressure gas barriers and preserve the set intra-abdominal pressure, in contrast to the trapdoor valves of conventional trocars. The ASM-Evac Tri-lumen Filter consists of one lumen for CO_2_ influx, one lumen for CO_2_ outflux to the IFS, and a third lumen for concurrent uninterrupted pressure assessment. Once the fixed pressure is reached, the CO_2_ flow is spontaneously reduced to 3 L/min, while preserving the fixed pressure. In group A, a 5 mm AirSeal® access port was used instead of a standard 5 mm port. Through the trial, laparoscopic equipment manufactured by Storz GmbH, Tuttlingen Germany, was employed. At the end of the surgery, the trocars were opened to release intra-abdominal CO_2_ and the abdomen was compressed by the surgeon’s hands to evacuate the residual gas. No local anesthetic was used in any patient.

### Outcomes and definitions

The primary endpoints were operative time, defined as the time from skin incision to closure of wound in minutes, and postoperative shoulder pain assessed by VAS (visual analogue pain scale). After surgery, patients were observed and interviewed for 2-day duration of shoulder pain evaluation by nurses. Postoperative pain was assessed in a double-blinded manner. Neither the patient, nor the assessor of shoulder pain, nor the postoperative caregivers were aware of the technique to which the patient was randomized. The 11-point VAS was used for assessment of the severity of shoulder pain (0 = none, 10 = severe) and scores were obtained at 1, 6, 12, 24 and 48 h after operation. All patients received standardized postoperative pain management. Anesthesia was terminated following extubation. Patients were closely monitored in the post-anesthetic care unit and then transferred to the surgical ward. A bolus of morphine sulfate (0.1 mg/kg) was administered intravenously (i.v.) after the operation. During postoperative period, all patients received 1 g Paracetamol and 1 g Metamizol i.v. every 6 h until 24 h postoperatively. Then pain was managed using oral Paracetamol and Metamizol on demand.

The secondary outcomes were length of hospital stay (days) and surgical side effects, including subcutaneous emphysema evaluation. Complications were graded according to the Clavien–Dindo classification [17].

### Statistical analysis

For statistical analysis, both a per-protocol (PP) and an intention-to-treat (ITT) analysis were used. It was defined that the PP analysis takes precedence in the evaluation of efficacy. There were no missing values for the two primary endpoints, hence the foreseen replacement procedure not coming into effect.

The randomized study had a confirmatory status (two parallel groups: AirSeal® CO2 pressure insufflation vs. standard CO2 pressure insufflation). A superiority approach was used. The following primary endpoints were defined (ranking in brackets): (1) duration of surgery [min] and (2) shoulder pain one day after surgery [VAS]. The type I error of one-sided 2.5% was maintained by a gatekeeping approach.

A sample size estimation based on pilot data for the prior ranking primary endpoint (time of surgery) resulted in a demand of at least 86 valid cases for efficacy in each group, group A (mean ± standard deviation): 74.8 ± 41.3 min and group S: 93.5 ± 41.3 min (one-sided type I error = 2.5%, type II error = 20%). Continuous variables are reported as medians and quartiles (in brackets). Categorical variables are expressed as counts and percentages (in brackets).

Metric variables and variables measured on ordinal scales were compared between the two groups by the exact Mann–Whitney U test, categorical variables by either the Fisher’s exact test or the exact chi-square test. Subgroup comparisons of time of surgery were performed by a non-parametric analysis of variance (Kruskal–Wallis one-way analysis of variance followed by Nemenyi's multiple comparisons).

Statistical analysis was performed using the open-source R statistical software package, version 3.0.1. The detailed sample size estimation and hypothesis have been published previously in our study protocol [14].

## Results

**Fig. 1:**
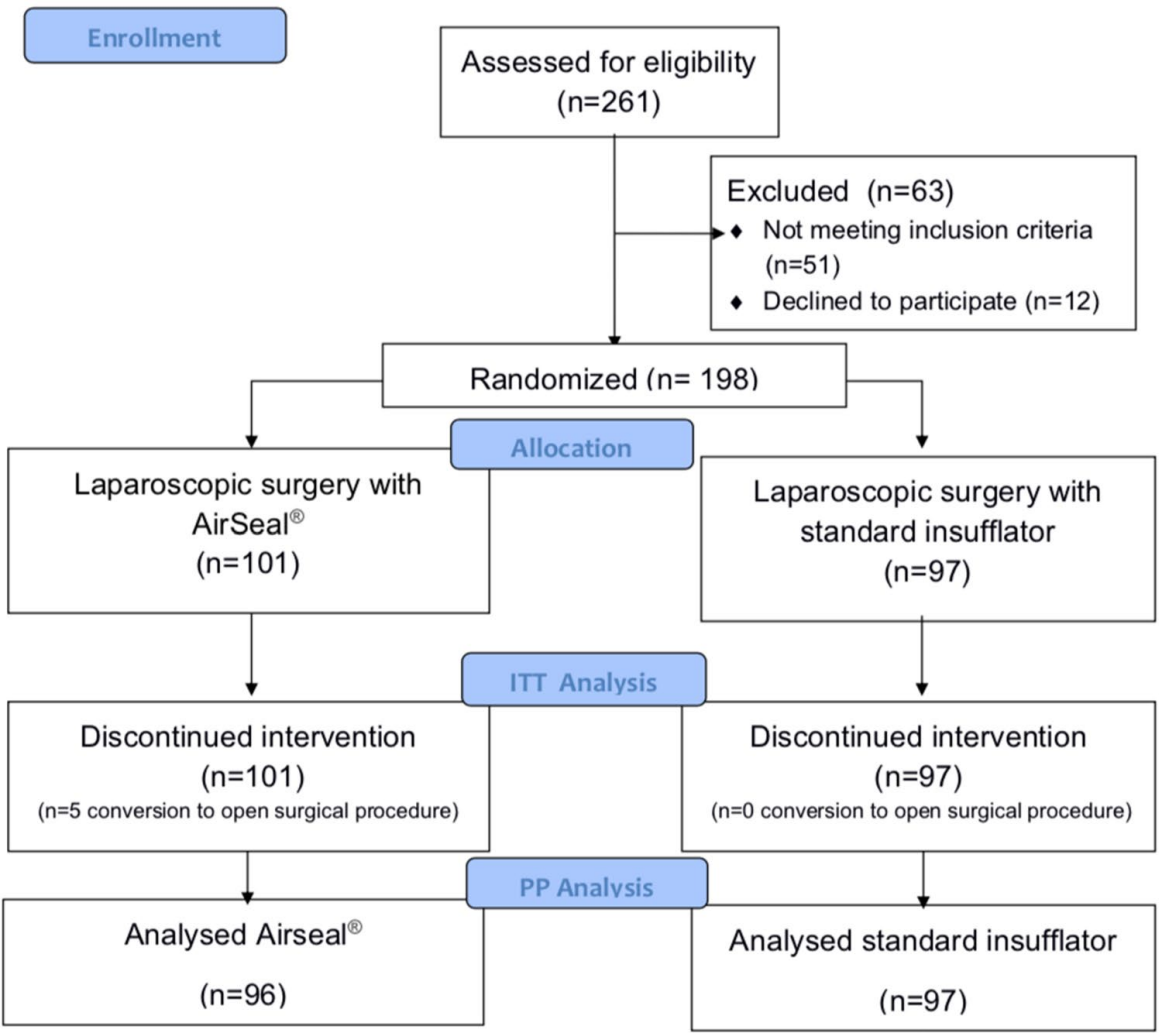
Consort diagram. *ITT* intention-to-treat, *PP* per-protocol

Between January 2013 and January 2014, a total of 261 surgical patients prior to elective laparoscopic cholecystectomy, sigmoid resection or laparoscopic hernia repair were screened for eligibility. Of these, 63 (24.1%) patients were excluded before randomization due to failure to meet the inclusion criteria or patients’ refusal to participate. The remaining 198 patients were allocated at random to the laparoscopic surgery with an AirSeal® CO_2_ pressure insufflator (Surgiquest Inc., Milford, USA) arm (group A, *n* = 101) or with a standard CO_2_ pressure insufflator (Olympus America Inc. UHI-3, Center Valley, PA, US) arm (group S, *n* = 97), and stratified according to type of operation. Five patients in group A and no patients in group S were converted to an open procedure due to technical difficulties in resection (*p* = 0.06). In 3 out of 5 patients, uncontrolled bleeding during sigmoid resection required laparotomy. In 2 of 5 cases, difficult anatomic findings during cholecystectomy led to conversion. These patients remained in the allocated group for intention-to-treat analysis. The flow of study participants through each stage of the trial is detailed in Fig. 1 in accordance with the CONSORT statement [18].

Baseline demographic characteristics and preoperative features are shown in Table 1. There were no statistically significant differences in sex, body mass index or age distribution between the two groups. Other baseline characteristics including history of surgery, comorbidity and ASA classification were neither statistically different between the groups.

**Table 1:** Baseline characteristics of patients

	AirSeal®	Standard	*P*
	(*n* = 96)	(*n* = 97)	
Age at surgery, median (IQR), y	54.0 ± 12.93	55.23 ± 13.2	0.12
Sex, n (%)			> 0.999
Male	44 (45.8)	44 (45.4)	
Female	52 (54.2)	53 (54.6)	
BMI, median (IQR), kg/m^2^	27.36 (24.64–30.95)	27.75 (24.84–30.08)	0.92
ASA grad, n (%)			0.75
I	36 (37.5)	35 (36.1)	
II	52 (54.2)	53 (54.6)	
III	8 (8.3)	9 (9.3)	
History of surgery, *n* (%)	32 (33.3)	28 (28.9)	0.67
Anticoagulants, *n* (%)	1 (1)	1 (1)	> 0.999
Antiplatelet agents, *n* (%)	9 (9.4)	11 (11.3)	0.18
Smoking history, *n* (%)	13 (13.5)	10 (10.3)	0.51
Diabetes, *n* (%)	4 (4.2)	6 (6.2)	0.78
Hypertension, *n* (%)	24 (25)	24 (24.7)	0.12
Procedures, *n* (%)			0.88
Lap. cholecystectomy	56 (58.3)	58 (59.8)	
Lap. sigmoid resection	6 (6.3)	8 (8.2)	
Lap. herniotomy			
Inguinal hernia unilateral	6 (6.3)	3 (3.1)	
Inguinal hernia bilateral	11 (11.5)	12 (12.4)	
Ventral hernia	17 (17.7)	16 (16.5)	

*ASA* American Society of Anesthesiologists, *BMI* body mass index, *lap*. laparoscopic, *IQR* interquartile range

**Fig. 2:**
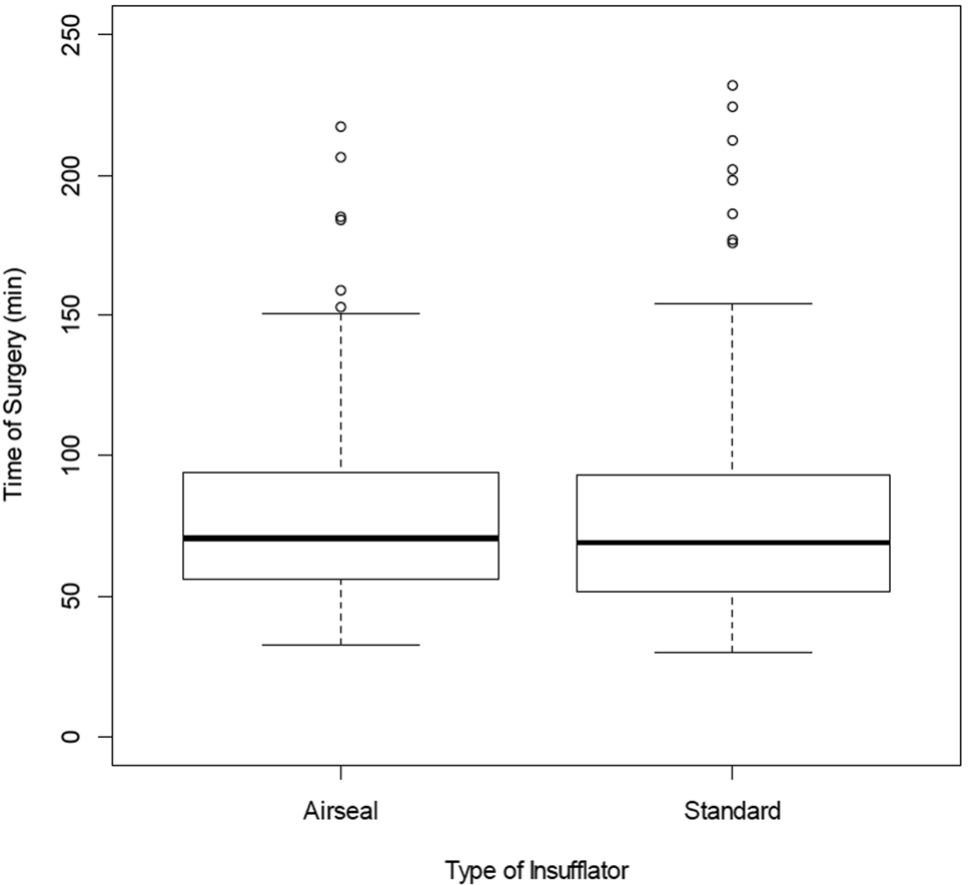
Difference in operative time between AirSeal® and standard insufflator

The primary study outcomes are summarized in Table 2. There was no significant difference in operative time (median [IQR]; 71 min [56–94] in group A vs. 69 min [52–93] in group S; *p* = 0.434) between the groups (Fig. 2). Even when stratifying between the different surgical procedures, there was no significant difference in the length of operating time detectable.

**Table 2:** Primary outcomes

	AirSeal®	Standard	*P*
	(*n* = 96)	(*n* = 97)	
Operating time, median (IQR), min	71 (56–94)	69 (52–93)	0.434
Lap. cholecystectomy	62 (54–86) (n = 56)	62.50 (48–79) (n = 58)	> 0.999
Lap. sigmoid resection	185 (176–206) (*n* = 6)	200 (181–218) (*n* = 8)	> 0.999
Lap. herniotomy	82 (60–102) (*n* = 34)	69 (59–85) (*n* = 31)	0.881
Postoperative shoulder pain, VAS	2 (0–4)	0 (0–3)	0.001

Data are presented as median (*IQR*, interquartile range); *min* minutes; *lap*. laparoscopic; *VAS* visual analogue pain scale

For laparoscopic cholecystectomy, the operative time was 61.50 min [53–85] for 56 patients in group A vs. 62.5 min [48–79] for 58 patients in group S; *p* > 0.999. In the laparoscopic herniotomy group, the operative time was 82 min [60–102] in 34 patients in group A vs. 69 min [59–85] in 31 patients in group S, p = 0.881. The mean operative time in laparoscopic sigmoid resection was 184.50 min [159–206] in 6 patients in group A vs. 200 min [181–218] in 8 patients in group S, *p* > 0.999, respectively, suggesting no significant differences between the groups.

**Fig. 3:**
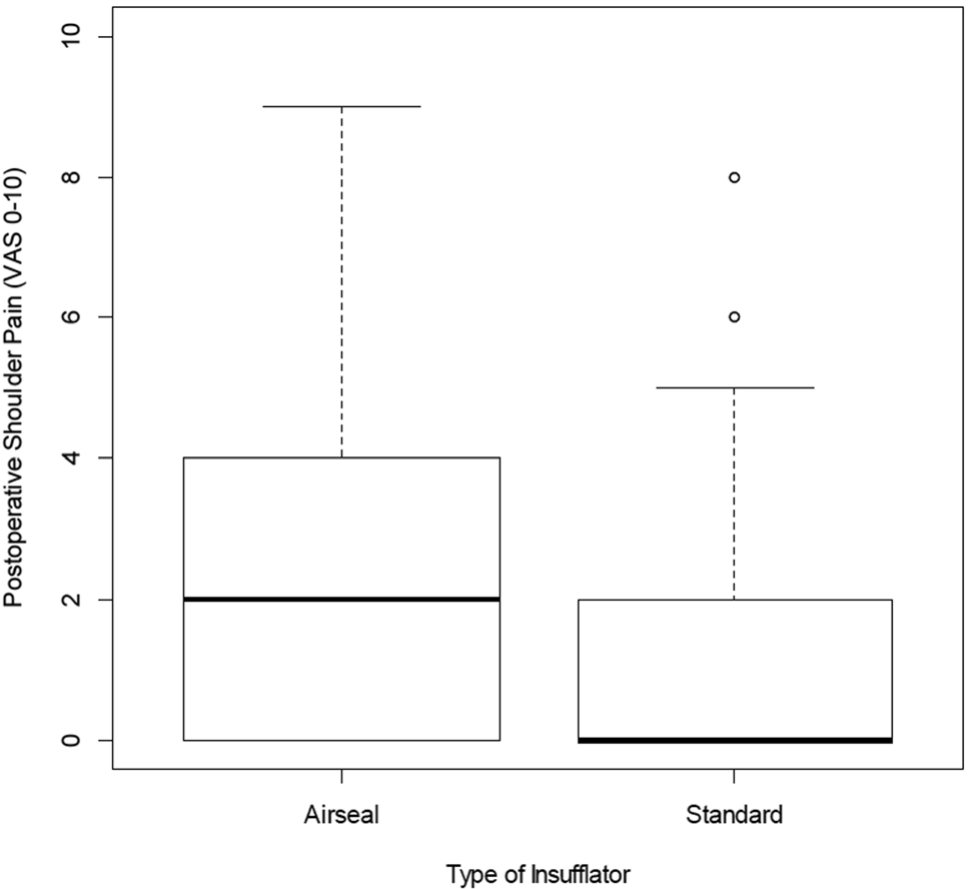
The severity of shoulder pain evaluated by visual analogue scale (0–10) for AirSeal® and standard insufflator

The proportion of patients reporting any level of postoperative shoulder pain during the first 48 h after operation was 90 of 193 patients (46.6%). Right-sided shoulder pain occurred in 37 patients, left-sided in 9 patients and pain on both sides in 44 patients. Incidence of shoulder pain was significantly lower within the standard group than in the AirSeal® group, 33 (34%) versus 56 (58.3%), *p* < 0.001. The VAS for pain was significantly lower in group S compared with group A (VAS 0 [0–3] vs. 2 [0–4]; *p* = 0.001) (Fig. 3). Shoulder pain was generally recorded to occur at 1–6 h and reached the peak at 12 h after surgery in both groups. Moreover, shoulder pain improved after 48 h postoperatively (Table 3).

**Table 3:** Postoperative shoulder pain

	AirSeal®	Standard	*P*
	(*n* = 96)	(*n* = 97)	
No shoulder pain at all, *n* (%)	40 (41.7)	64 (66)	< 0.001
1 h*	0.93 ± 1.21	0.96 ± 1.22	0.912
6 h*	3.6 (0–10)	3.1 (0–10)	0.643
12 h*	2.6 (0–10)	0.8 (0–8)	< 0.001
24 h*	2.7 (0–8)	1.5 (0–6)	< 0.001
48 h*	0.5 (0–6)	0.7 (0–7)	0.211

*Data are presented as median (*IQR* interquartile range) of *VAS* visual analogue pain scale (0–10)

Table 4 shows other surgical outcomes (length of hospital stay, drain insertion) and complications graded according to the Clavien–Dindo classification [17] that were not statistically different between the groups. All grade III classified complications in both groups were treated interventionally without general anesthesia, i.e., grade IIIa according to the modified Clavien–Dindo classification [17]. Subcutaneous emphysema was not observed in any patient. The difference in oral or intravenous analgesics taken between the 2 groups was not significant (*p* = 0.776 and 0.441, respectively). The most common postoperative complication was found to be surgical site infections (SSI) in 5 (5.2%) patients in arm A vs. in 2 (2.1%) patients in arm S. All of the SSIs were superficial and could be managed conservatively.

**Table 4:** Other surgical outcomes

	AirSeal®	Standard	P
	(*n* = 96)	(*n* = 97)	
Drain insertion, n (%)	10 (10.4)	12 (12.4)	0.821
Length of hospital stay, median (IQR), day	3.50 (3–4.5)	4 (3–5)	0.599
Blood transfusion	0	0	> 0.999
Total no. of complications	6	6	0.389
Subcutaneous emphysema	0	0	> 0.999
Severity of complications (Clavien–Dindo grade), *n* (%)			0.361
I	3 (3.13)	2 (2.06)	
II	1 (1.04)	0 (0)	
IIIa	2 (2.08)	4 (4.12)	
IIIb	0 (0)	0 (0)	
IV	0 (0)	0 (0)	
V	0 (0)	0 (0)	

*IQR* interquartile range

## Discussion

Laparoscopic surgery has played an important role in the advancement of modern surgery since it has first been applied in the diagnostic procedure [19]. Significant research work has been dedicated to identifying methods to reduce operative time and frequency and intensity of postoperative pain after laparoscopic surgery. It has been shown that low CO_2_ insufflation pressure reduces pain frequency and intensity after laparoscopy [6, 20]. However, a major disadvantage of using a low-pressure pneumoperitoneum is that exposure of the surgical field may be inadequate compared to standard pressure [21]. Adequate exposure of the surgical field is a prerequisite of safety, facilitates technical performance and reduces operative time. AirSeal® system reduces carbon dioxide absorption during laparoscopy when compared with standard trocars [22]. Moreover, standard CO_2_ insufflators often respond with delay to intraoperative pressure loss, thereby impeding adequate exposure due to collapse of the abdominal wall. By providing stable pneumoperitoneum and constant smoke evacuation, shorter operative time with AirSeal® system would be anticipated. Recently, closed pneumoperitoneum systems are recommended by several scientific societies to reduce the risk of infections via aerosols during laparoscopic surgery, especially in this period to control Covid-19 spreading.

This randomized controlled trial showed that using the AirSeal® system did not reduce operative time and was associated with higher risk of postoperative shoulder tip pain compared to standard CO_2_ insufflator. Even when stratifying between the different surgical procedures, there was no significant difference in the length of operating time. The outcome on operative time is in concordance with previous studies [23–26] demonstrating no difference in operative time between AirSeal® compared to the standard CO_2_ insufflator. This results should be interpreted with caution, regarding the fact that only one study [24] was a randomized controlled pilot trial with a small sample size (30 patients per group) and short operative time (median 26 min in AirSeal® vs. 30 min in standard insufflator group, p = 0.55).

Miyano et al. [23] prospectively compared AirSeal® (*n* = 18) to conventional pneumoperitoneum insufflator (*n* = 21) in pediatric laparoscopic appendectomy. In their study, intraperitoneal pressure was significantly lower during laparoscopic appendectomy with AirSeal®, but there was no significant difference in operative time (mean 76.2 min in standard group vs. 72.2 min in AirSeal®). These findings could be attributed to the observational nature of data and the small sample sizes, potentially resulting in a type I error. Furthermore, smoke production and minimal CO_2_ losses may be more prominent in pediatric patients with smaller intra-abdominal space compared to adults.

Two studies comparing AirSeal® with a standard Versaport trocar [25] and EndoClose™ [26] during minimally invasive urological surgery demonstrated no significant difference regarding overall operative time, although it was longer compared to studies showing significant operative time benefits for AirSeal® [22, 27]. The most possible explanation would be because of the heterogenous groups that comprised patients with different surgical approaches, the study designs and small sample sizes.

In one of the first studies that compared AirSeal® trocar with standard trocars in 51 patients undergoing laparoscopic renal surgery, Herati et al. [22] showed a significantly lower operative time in the AirSeal® group (124.13 vs 145.63 min, *p* = 0.47). The difference is likely attributed to the reduced need for surgical smoke evacuation with the AirSeal®. A retrospective review of patients undergoing robotic-assisted laparoscopic prostatectomy utilizing the AirSeal® system (*n* = 257) and conventional insufflation (*n* = 385) showed a significantly shorter mean operative time in the AirSeal® cohort (149.5 vs. 170.1 min, *p* < 0.0001) [28]. Annino et al. [27] also reported reduced operative time for AirSeal® in a prospective comparative study of robotic partial nephrectomy versus standard CO_2_ pressure insufflator (157 vs. 140 min, *p* = 0.03). In our pilot study, operative time was shorter in the AirSeal® group in patients who underwent a laparoscopic cholecystectomy. This observed difference may be due to unequally distributed comparison groups, with more teaching cases in the standard group and AirSeal® more frequent AirSeal® usage by experienced surgeons. However, all these studies [22, 27, 28] are limited by their non-randomized nature, predisposing a potential selection bias, but they all had a long operative time > 100 min in both groups.

However, in our small laparoscopic sigmoid resection subgroup there was a trend for shorter operative time in the AirSeal® system group. Major laparoscopic surgeries require drastically more use of electrosurgery compared to less complex surgeries. The absence of a significant difference in operative times in our study could be attributed to the fact that mainly short operative time procedures were evaluated. It is necessary to evaluate the outcome of AirSeal® in randomized controlled trials on complex procedures, with longer operating time and higher smoke production, such as low anterior rectum resection, esophagectomy or transanal total mesorectal excision (TaTME). Such procedures might further benefit from AirSeal® due to a possibly improved microperfusion profile compared to standard insufflator, as suggested by an animal study [29].

In our study, we used the visual analog scale, which was feasible for all our patients. The AirSeal® group rated shoulder pain higher, and this result differed from the result obtained and published by Sroussi et al. [24] where the shoulder tip pain in AirSeal® group was lower compared to standard pressure pneumoperitoneum. However, the AirSeal® group was a low-pressure group with 7 mmHg compared to control group with 15 mmHg pneumoperitoneum. In our study, the intra-abdominal pressure was set at 12 mmHg in both groups. Low pressure (< 10 mmHg) has in several studies been associated with a significant reduction in both incidence and severity of shoulder pain [8, 30–33]. Additionally, the study design included different types of surgical procedures stratifying 30 patients. The result may be attributed to insufficient sample and lack of power to evaluate this variable in this study. In a RCT using an equal allocation ratio across 4 arms (standard insufflation vs. valveless insufflation with an intra-abdominal pressure of 10 mmHg or 15 mmHg) in 33 patients per group, no difference in patients postoperative shoulder pain was reported [34].

On the basis of our data we cannot explain that pneumoperitoneum with AirSeal® is causing more postoperative shoulder tip pain. We postulate that it may be a result of the constant pressure pneumoperitoneum compared to the standard insufflator where the pressure changes during some maneuvers such as suction. Apart from postoperative pain, there were no significant intraoperative or postoperative complications in either group.

In the present trial, we have not included cost assessment; the additional factor of AirSeal® system consumables has to be taken into account. There are no trials reporting the cost on AirSeal® usage. A cost reduction as a result of shorter duration of complex surgical procedures using AirSeal® may be hypothesized, although relevant evidence is missing.

Although there was no difference in operative time in our study, participating surgeons reported improved visualization of the operative field with AirSeal®, especially in laparoscopic colorectal surgery. This is also an important feedback that may impact the decision to use AirSeal® in more complex surgical procedures. Maduueke-Laveaux et al. [34] assessed surgeon’s visualization of the operative field with valveless versus standard insufflation system in a randomized controlled trial. Surgeons reported improved visualization of the operative field using valveless insufflation system over standard insufflation (p < 0.001), and this was most significant when performing complex robotic gynecologic surgeries that require more electrocautery. Further trials may detect clinically significant differences for specific procedures.

## Limitations of the study

This RCT considered three different types of laparoscopic procedures, which require different patient positioning, use of electrocautery and extent of dissection. The study was powered to detect differences across procedures; however, the outcomes may not be applicable to individual procedures. Nevertheless, stratified randomization suggests that AirSeal® does not provide advantages across a variety of procedures. The study lacked power and adequate sample size in the laparoscopic sigmoid resection group where operation time was longer. Evaluation of visualization of the operative field reviewed by a blinded surgeon was not done that might have an impact on operative time. Blinding of the surgeons could not be applied due to logistical challenges and performance bias can therefore not be excluded.

This study was however powered to detect differences in the primary outcome measure and operative time. Additionally, clinically relevant secondary outcomes were evaluated, such as postoperative shoulder tip pain and subcutaneous emphysema. Furthermore, it adhered to methodological principles of RCTs, including blinding of participants and personnel.

## Conclusion

Application of the AirSeal® system did not reduce operative time and was associated with a higher postoperative shoulder tip pain compared to standard CO_2_ insufflator for short elective surgeries.
